# Interaction of Natural Compounds in Licorice and Turmeric with HIV-NCp7 Zinc Finger Domain: Potential Relevance to the Mechanism of Antiviral Activity

**DOI:** 10.3390/molecules26123563

**Published:** 2021-06-10

**Authors:** Runjing Wang, Yinyu Wei, Meiqin Wang, Pan Yan, Hongliang Jiang, Zhifeng Du

**Affiliations:** 1 Tongji School of Pharmacy, Huazhong University of Science and Technology, Wuhan 430030, China; wangrjcn@foxmail.com (R.W.); weinyinyu@163.com (Y.W.); wangmeiqin@hust.edu.cn (M.W.); panyan_science@163.com (P.Y.); jianghongliang@hust.edu.cn (H.J.); 2 Ningxia Pharmaceutical Inspection and Research Institute, Yinchuan 750001, China

**Keywords:** zinc fingers, fluorescence, mass spectrometry, anti-HIV, natural product

## Abstract

Nucleocapsid proteins (NCp) are zinc finger (ZF) proteins, and they play a central role in HIV virus replication, mainly by interacting with nucleic acids. Therefore, they are potential targets for anti-HIV therapy. Natural products have been shown to be able to inhibit HIV, such as turmeric and licorice, which is widely used in traditional Chinese medicine. Liquiritin (LQ), isoliquiritin (ILQ), glycyrrhizic acid (GL), glycyrrhetinic acid (GA) and curcumin (CUR), which were the major active components, were herein chosen to study their interactions with HIV-NCp7 C-terminal zinc finger, aiming to find the potential active compounds and reveal the mechanism involved. The stacking interaction between NCp7 tryptophan and natural compounds was evaluated by fluorescence. To elucidate the binding mode, mass spectrometry was used to characterize the reaction mixture between zinc finger proteins and active compounds. Subsequently, circular dichroism (CD) spectroscopy and molecular docking were used to validate and reveal the binding mode from a structural perspective. The results showed that ILQ has the strongest binding ability among the tested compounds, followed by curcumin, and the interaction between ILQ and the NCp7 zinc finger peptide was mediated by a noncovalent interaction. This study provided a scientific basis for the antiviral activity of turmeric and licorice.

## 1. Introduction

Zinc finger proteins are proteins that use zinc as the coordination center, and the coordination is between zinc and cysteine or histidine from peptides [1]. The nucleocapsid protein, a zinc finger protein, is particularly important, as it is highly conserved and plays a central role in HIV virus replication, mainly by interacting with nucleic acids. Therefore, it is one of the drug targets for HIV therapy [2,3,4], and it is complementary to drugs such as protease inhibitors and nucleoside analogs aimed at other targets of the viral life cycle [4], which cause drug resistance easily [5,6,7]. The presence of the W37 residue in the nucleocapsid protein has been revealed as a key feature for molecular recognition from the zinc finger toward oligonucleotides [8,9].

Natural products have been shown to be able to inhibit HIV [6,10,11]. An example is licorice, the roots and rhizomes of *Glycyrrhiza uralensis*, *Glycyrrhiza inflata* and *Glycyrrhiza glabra* [12,13]. Glycyrrhizin (GL), the aqueous extract from licorice root, is effective in preventing the progression of disease in a mouse model [14,15]. The treatment comprised of GL, glycine and cysteine was found to be effective in the treatment of HIV-infected patients, as shown by the improvement in the clinical symptoms and immunological and liver functions [16]. Curcumin (CUR), a natural phytochemical derived from turmeric, also showed HIV inhibition activity [17,18,19,20,21]. Curcumin has no toxicity, and it was in clinical trials for AIDS patients [22]. The detailed mechanism underlying this is still unclear.

Small molecules from natural products can form covalent or noncovalent complexes with targets, accompanied by inhibiting the activity of enzymes or proteins, which is typically the action mode of natural compounds [23,24,25]. Spectrometric and spectroscopic methods are useful in revealing the interactions between small molecules and their targets [26,27,28,29]. As reported previously [6], nordihydroguaiaretic acid was found to inhibit nucleocapsid (NC) and the replication of wild-type and drug-resistant HIV-1 strains in the low micromolar range with moderate cytotoxicity. The mechanism of action was elucidated by nuclear magnetic resonance, mass spectrometry, fluorescence spectroscopy and molecular modeling. Nordihydroguaiaretic acid was found to act through a dual mechanism of action never highlighted before for NC inhibitors (NCIs)—namely, noncovalent binding—resulting in the inhibition of the nucleic acid chaperone properties of NC, followed by the chemical oxidation of nordihydroguaiaretic acid inducing the inactivation of the protein.

**Figure 1:**
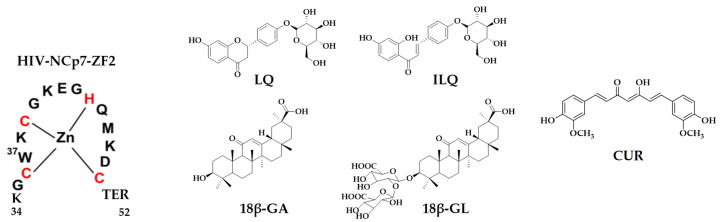
Sequence of the C-terminal zinc finger of HIV-NCp7 and the chemical structures of the compounds used in this study.

Tryptophan (Trp) of the HIV-NCp7 zinc finger has a fluorescence emission, while the stacking interaction quenches that fluorescence. A previous study suggested a good correlation between tryptophan quenching and the inhibition of NCp7-nucleic acid binding [30]. Therefore, natural compounds CUR, liquiritin (LQ), isoliquiritin (ILQ), glycyrrhizic acid (GL) and glycyrrhetinic acid (GA), the major components of turmeric and licorice, were selected in this study, and the binding between the tryptophan of NCp7 and natural compounds was evaluated by fluorescence. The binding mode between the inhibitors and targets was studied by mass spectrometry. CD spectroscopy and molecular docking were used to validate and reveal the binding mode from a structural perspective. The chemical structures of CUR, LQ, ILQ, GL and GA are shown in Figure 1.

## 2. Results and Discussion

### 2.1. Characterization of Zinc Finger by CD Spectroscopy

**Figure 2:**
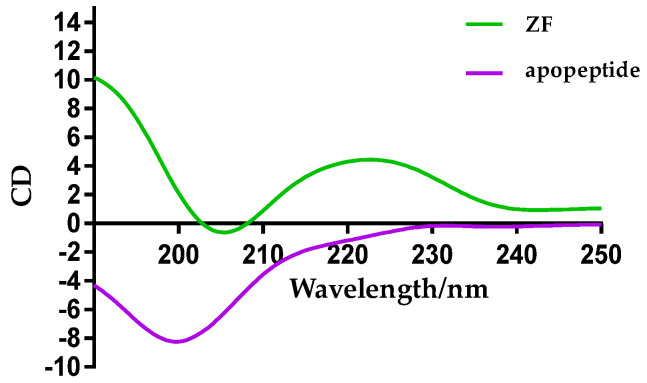
CD spectra of 100-μM ZF and 100-μM apopeptide.

Circular dichroism gives information about the secondary structure of the proteins. It is a useful tool to characterize the formation of a zinc finger, as the CD spectrum of NCp7 displays a positive maximum around 220 nm. As we can see from Figure 2, the profiles of the apopeptide and zinc finger (ZF) peptide are different, according to the literature [31]. Based on the CD spectrum, the formation of the zinc finger structure was confirmed.

### 2.2. Evaluation of the Binding between NCp7 ZF and Natural Compounds by Fluorescence Assay

A library of approximately 2000 small molecules from the NCI diversity set suggested a good correlation between tryptophan quenching and the inhibition of NCp7-nucleic acid binding for fluorescein-based compounds [30]. Therefore, we evaluated the NCp7 inhibitors from natural products by fluorescence. The fluorescence quenching of Trp was measured in the presence of natural compounds in order to compare the association affinity [32]. It can be a good indicator of the strength for an interaction [33].

Firstly, the fluorescence of LQ, ILQ, 18β-GA and 18β-GL and CUR was measured to exclude interference. A 5-mM concentration was prepared in DMSO. As shown in Figure S1, ILQ, GL, GA and CUR had no fluorescence under the tested conditions, while LQ had observable fluorescence. Therefore, the fluorescence of most compounds will not interfere with the measurements, except for LQ.

**Figure 3:**
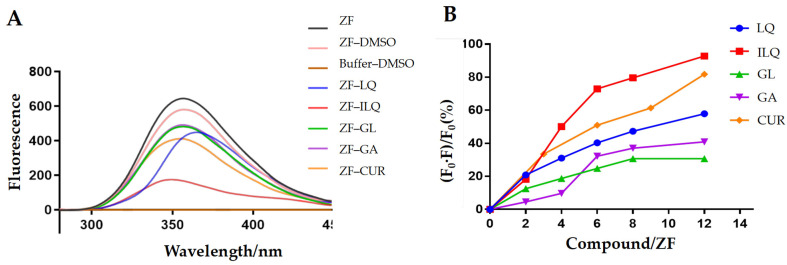
(**A**) The fluorescence emission spectra of ZF upon incubation with different natural compounds. Spectra were recorded for 5-µM ZF after incubation with 30-µM complexes. (**B**) Fluorescence quenching ratio of ZF upon adding different ratios of natural compounds ((ZF)/(compounds)). Titration was performed with 5-µM ZF at RT. F represents the fluorescence intensity of ZF at 356 nm during titration, and F0 is the fluorescence intensity of ZF only at 356 nm.

Subsequently, the emission spectrum of 5-µM zinc finger at pH 7.4 (50-mM NaCl in 20-mM tris buffer) was acquired. The emission spectrum of 5-µM zinc finger in a buffer with DMSO was also acquired to exclude the influence of an organic solvent. As we can see from Figure 3A, the DMSO has no influence on the assay. The emission spectrum of the buffer with DMSO was also acquired to exclude the fluorescence from the background. As we can see from Figure 3A, the buffer with DMSO has no fluorescence. Therefore, the DMSO and buffer will not influence the fluorescence of zinc finger during the assay.

Then, the emission spectrum of 5-µM zinc finger incubated with 30-µM compounds was acquired. As we can see from Figure 3A, under the same molar ratio, all the compounds can quench the fluorescence of zinc finger to some extent. The strongest quenching comes from ILQ, then CUR, and the same extent of quenching was observed for GL and GA. LQ has a similar extent of quenching; however, its own fluorescence interferes with the measurement of quenching, as we can observe by the shift of the emission spectrum.

To compare the binding strengths between the natural compounds and zinc finger, 5-µM zinc finger was titrated with ILQ, LQ, GL, GA and CUR at molar ratios (ZF/compounds) from 1 to 12. The emission maximum (356 nm) was measured immediately after each titration. As we can see from Figure S2 and Figure 3B, the extent of quenching was consistent with that of 6:1 for different compounds. At 12 to 1, ILQ and CUR quenched most of the fluorescence. Therefore, they were screened as the strongest binders among the tested compounds, which was consistent with the in vitro anti-HIV activity of the ILQ analog and CUR [20,34].

Subsequently, the binding kinetics between ZF and CUR/ILQ were studied. The fluorescence of ZF quenched by six equivalents of CUR/ILQ was measured at different time points. As shown in Figure S3, the binding was immediate, and the fluorescence remained constant even after 12 h; therefore, we proposed the noncovalent binding mode for those two compounds with zinc finger. To validate the binding mode of ILQ with zinc finger, mass spectrometry and CD spectroscopy were utilized.

### 2.3. Elucidation of the Binding Mode between NCp7 ZF and Natural Compounds by Mass Spectrometry

Mass spectrometry has been successfully used to study the noncovalent and covalent interactions between the zinc finger and small molecules [2,6,31,35,36]. In this work, the reaction mixture of ZF and the active compounds was characterized by electrospray ionization-mass spectrometry (ESI-MS). Firstly, ILQ was dissolved in methanol with a concentration of 5 mM; then, it was incubated with the zinc finger peptide at the 1:3 and 1:6 molar ratios, respectively. The reaction mixture was diluted with methanol water to obtain a final concentration of 10 µM; subsequently, an ESI-MS analysis was carried out to monitor the reaction. For the 1:3 molar ratio, the reaction mixture was monitored at 15 min, 2 h and 5 h, respectively.

**Figure 4:**
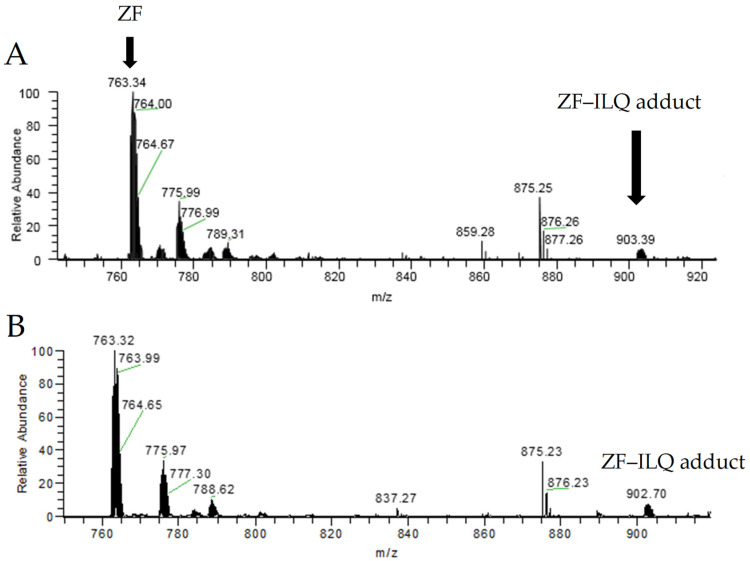
ESI-MS spectra of the reaction mixture between ZF and ILQ. (**A**) 10 μM of zinc finger were incubated with 30-μM ILQ for 15 min. (**B**) 10 μM of zinc finger were incubated with 60-μM ILQ for 15 min.

The mass spectra of the reaction mixture of the zinc finger and ILQ are shown in Figure 4, and the species at the 1:3 or 1:6 molar ratios were similar when the reaction time was 15 min. The zinc finger was observed as the major species, and the noncovalent adduct was the minor species. The disagreement between strong fluorescence quenching and a low ratio of noncovalent adduct was due to the dissociation of the noncovalent adduct during the mass spectrometric analysis. The observation of noncovalent species and free zinc finger indicates the noncovalent binding mode between ILQ and the zinc finger at the early time points. Similarly, we observed noncovalent-binding species for CUR, as shown in Figures S6 and S8.

To monitor the species formed at different time points, the reaction mixture was analyzed by ESI-MS at 1:3 for 15 min, 2h and 5 h, respectively. As shown in Figure S4, the species formed at different time points are the same, and the relative intensities of the important species are similar. These results were consistent with the kinetic results from the fluorescence analysis. Therefore, the immediate noncovalent binding mode between ILQ and zinc finger was confirmed. Similar results were observed for CUR, as shown in Figure S7.

The species observed were identified by high-resolution mass spectrometry. The theoretical and experimental spectra are shown in Figures S5 and S8, and the *m/z* and isotope pattern are consistent between the theoretical and experimental results. The observed species are summarized in Table 1. The major species are zinc finger and the noncovalent adduct of zinc finger with ILQ, which is also the case for CUR, as shown in Table 1. At the same time, a covalent adduct of an oxidized peptide with ILQ was also observed as a minor species, while the covalent species was not observed for CUR. These results showed that noncovalent recognition will gradually induce covalent binding.

Thereafter, we suggested that the possible mechanism was as follows: firstly, the molecule recognizes and binds NC noncovalently; in the second step, the ejection of zinc from the noncovalent adduct deprotects the cysteine amino acid in zinc finger; in the final step, the oxidation of cysteine is induced by the unsaturated keto group of ILQ. For CUR, its activity is lower than that of ILQ; thus, we did not observe the dissociation of Zn. The inhibiting activity of ILQ towards the binding between the zinc finger peptide and nuclear acid should be tested in the future.

In summary, based on the mass spectrometric results, it is clear that ILQ initially binds to the NCp7 C-terminal zinc finger peptide by the noncovalent mode, followed by covalent binding. The possible mechanism was elucidated herein.

### 2.4. Structural Information of the Binding from CD Spectroscopy and Molecular Docking

**Figure 5:**
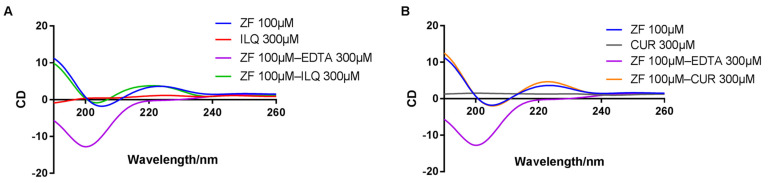
Characterization of the ZF structure upon ILQ or CUR binding. (**A**) CD spectra of ZF at t = 2 h from the incubation with ILQ at 3:1 ([ILQ]/[ZF]) (**B**) CD spectra of ZF after the incubation with CUR for 2 h at 3:1 ([CUR]/[ZF]).

Circular dichroism spectroscopy has been widely used to study the disturbance of zinc finger protein conformation by small molecules [2,31,35]. The CD spectrum of the C-terminal ZF of HIV-NCp7 is characterized by a positive band at around 220 nm and a negative band at 195–200 nm [31,37]. The loss of zinc from the ZF results in a decrease in the positive band and a significant increase in the intensity of the negative band. As shown in Figure 5, the CD profiles of the zinc finger protein and reaction mixture are similar. Incubation with CUR or ILQ for 2 h does not change the secondary structure of ZF. Therefore, the noncovalent binding mode was also validated by CD spectra.

**Figure 6:**
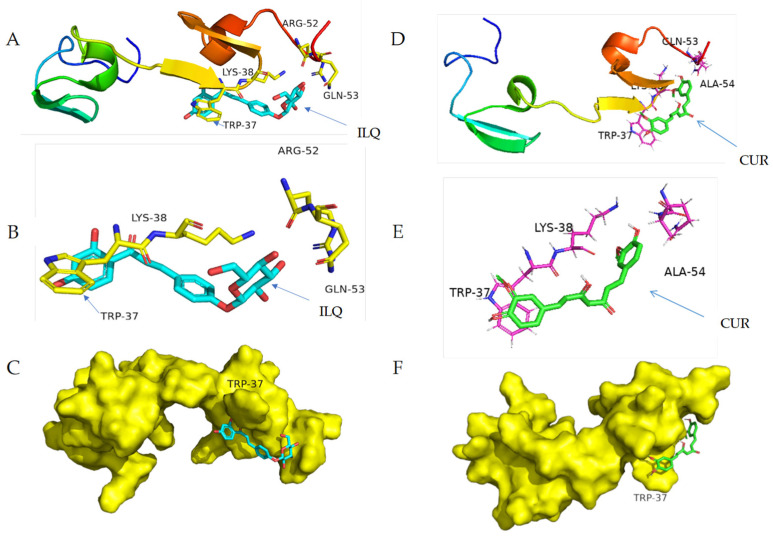
Molecular docking of HIV-NCp7 ZF with ILQ and CUR: (**A**,**D**) overview, (**B**,**E**) zoom in of the stacking region and (**C**,**F**) surface of the protein with ILQ and CUR, respectively.

To obtain the structural details of noncovalent binding, molecular docking was performed for the strongest binder. AutoDock is an automated docking tool. It is designed to predict how small molecules, such as substrates or drug candidates, bind to a receptor of a known 3D structure. AutoDock Vina software was used herein to show the structure of the complex. As shown in Figure 6, molecular docking revealed edge-to-face π-π stacking between the protein tryptophan and ILQ aromatic ring, which was consistent with the interaction mode of the nucleic acid bases and other aromatic compounds [2,6,32,38]. For CUR, face-to-face π-π stacking was observed between the protein tryptophan and CUR aromatic ring. The overview and zoom in for the molecule docking of HIV-NCp7 ZF with ILQ and CUR are displayed in Figure 6A,B,D,E, respectively. To give a clear view of the binding, the surface of HIV-NCp7 is displayed with ILQ and CUR attached in Figure 6C,F, respectively.

## 3. Materials and Methods

### 3.1. Reagents and Equipment

The pure compounds of LQ, ILQ, GL, GA and curcumin were bought from the National Pharmaceutical Engineering Center for Solid Preparations of Chinese Herbal Medicine (Nanchang, China). The NCp7 C-terminal peptide sequence (KGCWKCGKEGHQMKDCTER) was purchased from China peptide Co., Ltd. (Shanghai, China). All the other reagents were purchased from Sigma Aldrich, USA and used without further purification.

Fluorescence studies were recorded on a PerkinElmer fluorometer. CD spectra were obtained with a JASCO J-1500 Spectropolarimeter (Jasco Corp., Tokyo, Japan) in the wavelength range of 190-250 nm, using a cuvette with a path length of 0.1 cm. Mass spectrometry experiments were carried out on an Orbitrap Velos from the Thermo Electron Corporation (Waltham, MA, USA).

### 3.2. Methods and Procedure

#### 3.2.1. Preparation of Zinc Finger Protein

The preparation of the zinc finger protein followed the published methods [31]. The apopeptide was dissolved in deionized water at a concentration of 1 mM. Zinc acetate (1.2 eq.) was added to the solution, and the pH was adjusted to 7.0 using NH_3_·H_2_O. The zinc finger solution was incubated for 2 h at 37 °C before recording any experiments. Secondary structure of the ZF was characterized by CD spectroscopy.

#### 3.2.2. Fluorescence Assay

The experiments were carried out in 20-mM Tris buffer with 50-mM NaCl at pH 7.4. 5 μM of zinc finger were titrated with pure compounds at molar ratios of 1–12 (ZF to drug). The fluorescence of 12 molar equivalents of natural compounds was monitored in the same buffer. Samples were irradiated with 280 nm of light, and spectra were recorded from 280 to 450 nm with a scan rate of 600 nm/min at 25 °C. The slit was 4.0 nm. The emission maximum (356 nm) was measured after each titration. Please see Supplementary materials for more details.

#### 3.2.3. Mass Spectrometry

For the mass spectrometry experiments, the zinc finger samples were prepared in an aqueous solvent at 1 mM and incubated with an appropriate concentration of natural compounds in methanol. The reaction solutions were diluted with methanol/water (1:1 *v*/*v*) to ~10 μM. Experiments were carried out in the positive mode. Samples were directly infused at a flow rate of 1 μL/min using a source voltage of 3.5 kV. The source temperature was maintained at 280 °C throughout. The data was processed by Xcalibur software.

#### 3.2.4. Docking

For molecular docking, AutoDock Vina software was used [39]. The NMR structure of HIV-NCp7 was downloaded from the PDB (PDB code: 1MFS). The structures of ILQ and CUR were generated by ChemDraw and OpenBabel. The charge was automatically added into ILQ and CUR, and the rotation of the bond was defined by the AutoTors program in AutoDock. Before docking, polar hydrogen atoms were added to the HIV-NCp7 protein; then, the Kollman charge was calculated. The docked grid box was a cubic 3D space with a size of 20 Å × 20 Å × 20 Å and a grid interval of 1 Å.

## 4. Conclusions

Turmeric and licorice are widely used in traditional Chinese medicine, and they have been shown to be able to inhibit HIV. However, the detailed mechanism how is still unclear. The NCp ZF protein plays a central role in HIV virus replication, and it can stack with aromatic compounds. Since liquiritin, isoliquiritin, glycyrrhizic acid, glycyrrhetinic acid and curcumin are the major active components of licorice and turmeric, the binding between NCp7 and those natural compounds was studied herein. Fluorescence results showed that ILQ has the strongest binding ability among the tested compounds, followed by curcumin. The mass spectrometry analysis showed that the interaction between ILQ/CUR and the NCp7 zinc finger peptide was mediated by a noncovalent interaction, which was consistent with the CD spectra results. Molecule docking revealed the edge-to-face and face-to-face π-π stacking between the protein tryptophan and ILQ and CUR aromatic rings. This study provided scientific evidence and hints of the antiviral activity of turmeric and licorice.

## Figures and Tables

**Table 1:** Species observed in the ESI-MS spectra (charge +3).

Species	Formula	Mono Observed *m/z*
ZF	C_90_H_144_N_30_O_28_S_4_Zn	762.62
ZF–ILQ adduct	C_111_H_166_N_30_O_37_S_4_Zn	902.02
Oxipeptide–ILQ	C_111_H_166_N_30_O_37_S_4_	880.71
ZF–CUR adduct	C_111_H_164_N_30_O_37_S_4_Zn	885.35

## Data Availability

The data presented in this study are available on request from the corresponding author.

## Supplementary Materials

The following are available online: Figure S1. The fluorescence emission spectra of different complexes (30 μM). Figure S2. Fluorescence emission spectra of ZF after adding increasing amounts of LQ, ILQ, GL, GA and CUR, respectively. Figure S3. The fluorescence emission spectra of ZF upon incubation with CUR or ILQ at different time intervals. Spectra were recorded with 5-µM ZF incubated with 30-µM CUR/ILQ for 10 min, 30 min, 1 h and 12 h, respectively. Figure S4. ESI-MS spectra of the reaction mixture between ZF and ILQ. The selected region of the spectra showed 3+ charged peaks. The reaction was performed on 10-μM ZF with 30-μM ILQ. The spectra were recorded after incubation for 15 min (A), 2 h (B) and 5 h(C), respectively. Figure S5. Species observed in the ESI-MS spectrum for the reaction mixture between ZF and ILQ. Observed (A,C,E) and calculated (B,D,F) mass spectra of the species. Figure S6. ESI-MS spectra of the reaction mixture between ZF and CUR. 10 μM of zinc finger were incubated with 60-μM CUR for 15 min (top). 10 μM of zinc finger were incubated with 30-μM CUR for 15 min (bottom). Figure S7. ESI-MS spectra of the reaction mixture between ZF and CUR. The selected region of the spectra showed 3+ charged peaks. The reaction was performed on 10-μM ZF with 60-μM CUR. The spectra were recorded after incubation for 15 min, 2h and 5 h, respectively. Figure S8. The species observed in the ESI-MS spectrum for the reaction mixture between ZF and CUR. Observed (left) and calculated (right) mass spectra of the species.

## Sample Availability

Samples of the compounds are available from the authors.

## Abbreviations

ZF	zinc finger
LQ	liquiritin
ILQ	isoliquiritin
GL	glycyrrhizic acid
GA	glycyrrhetinic acid
CUR	curcumin
HIV-NCp7	the human immunodeficiency virus nucleocapsid protein
NCIs	NC inhibitors
CD	circular dichroism
ESI-MS	electrospray ionization-mass spectrometer
