# Supplementary material for: Interaction of Natural Compounds in Licorice and Turmeric with HIV-NCp7 Zinc Finger Domain: Potential Relevance to the Mechanism of Antiviral Activity
Source: Molecules. 2021 Jun 10;26(12):3563. doi: 10.3390/molecules26123563 (PMC8230585; doi:10.3390/molecules26123563)
Supplement: Supplementary file 1 [file molecules-26-03563-s001.zip › molecules-1194926-supplementary.pdf]

Supplementary materials

Interaction of Natural Compounds in Licorice and Turmeric with HIV-NCp7 Zinc Finger Domain: Potential Relevance to the Mechanism of Antiviral Activity

Runjing Wang [1,2,†], Yinyu Wei [1,†], Meiqin Wang [1], Pan Yan [1], Hongliang Jiang [1] and Zhifeng Du [1,*]

[1] Tongji School of Pharmacy, Huazhong University of Science and Technology, Wuhan 430030, China; wangrjcn@foxmail.com (R.W.); weinyinyu@163.com (Y.W.); wangmeiqin@hust.edu.cn (M.W.); panyan_science@163.com (P.Y.); jianghongliang@hust.edu.cn (H.J.)

[2] Ningxia Pharmaceutical Inspection and Research Institute, Yinchuan 750001, China

* Correspondence: duzhifeng@hust.edu.cn

† These authors contributed equally to this work.

LIST OF CONTENTS

Figure S1. The fluorescence emission spectra of different complexes (30μM).

Figure S2. Fluorescence emission spectra of ZF after adding increasing amount of LQ, ILQ, GL, GA and Cur, respectively.

Figure S3. The fluorescence emission spectra of ZF upon incubation with Cur (top) and ILQ (bottom) at different time intervals. Spectra were recorded with 5 μM ZF incubated with 30 μM Cur/ILQ for 10 min, 30 min, 1h and 12h, respectively.

Figure S4. ESI-MS spectra of the reaction mixture between ZF and ILQ. The selected region of the spectra shows 3+ charged peaks. The reaction was performed on 10 μM ZF with 30 μM ILQ. The spectra were recorded after incubation for 15 min (A), 2h(B) and 5 h(C), respectively.

Figure S5. Species observed in ESI-MS spectrum for the reaction mixture between ZF and ILQ. Observed (A, C, E) and calculated (B, D, F) mass spectra of the species.

Figure S6. ESI-MS spectra of the reaction mixture between ZF and CUR. 10 μM ZF was incubated with 60 μM CUR for 15 min (top) .10 μM ZF was incubated with 30 μM CUR for 15 min (bottom).

Figure S7. ESI-MS spectra of the reaction mixture between ZF and CUR. The selected region of the spectra shows 3+ charged peaks. The reaction was performed on 10 μM

ZF with 60 μM CUR. The spectra were recorded after incubation for 15 min, 2h and 5 h respectively.

Figure S8. Species observed in ESI-MS spectrum for the reaction mixture between ZF and CUR. Observed (left) and calculated (right) mass spectra of the species.

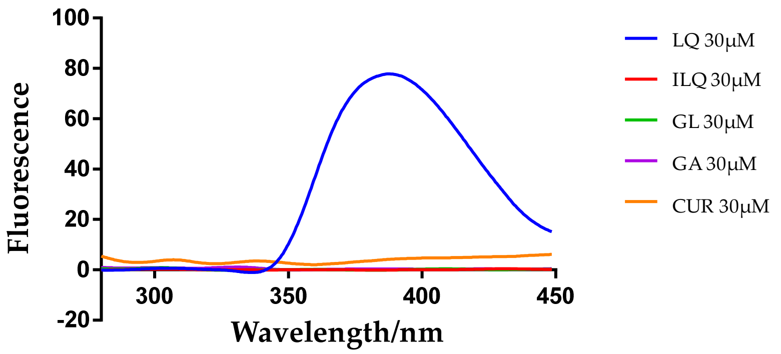

Figure S1. The fluorescence emission spectra of different complexes (30μM).

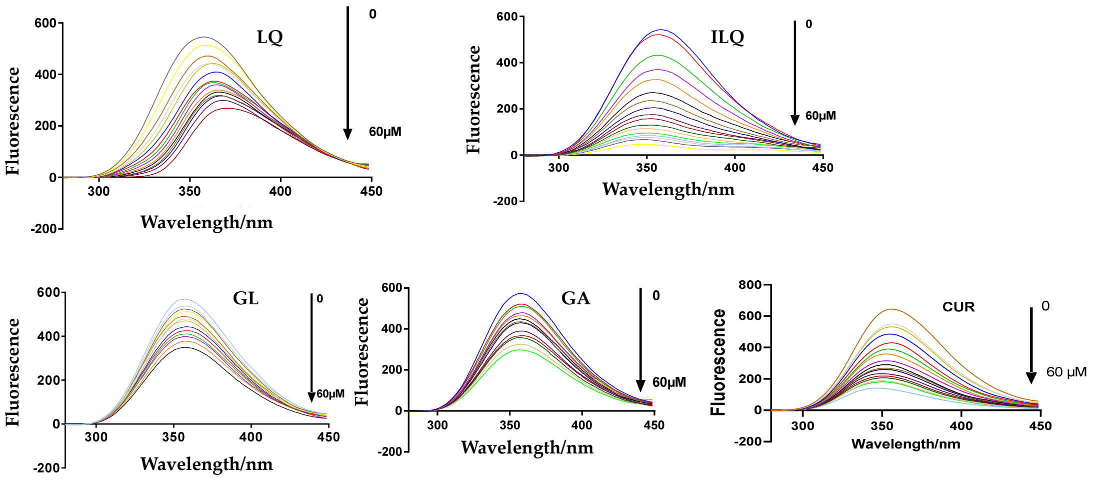

Figure S2. Fluorescence emission spectra of ZF after adding increasing amount of LQ, ILQ, GL, GA and Cur, respectively.

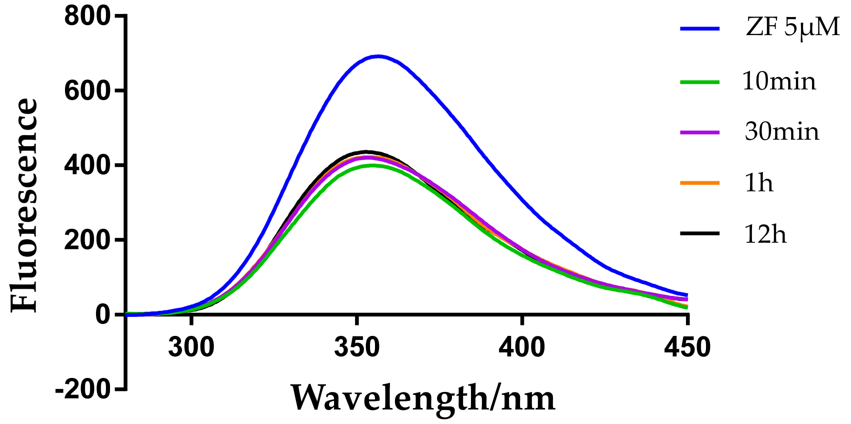

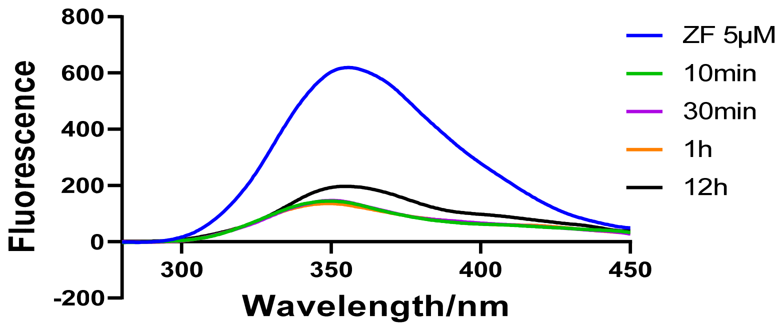

Figure S3. The fluorescence emission spectra of ZF upon incubation with Cur (top) and ILQ (bottom) at different time intervals. Spectra were recorded with 5 μM ZF incubated with 30 μM Cur/ILQ for 10 min, 30 min, 1h and 12h, respectively.

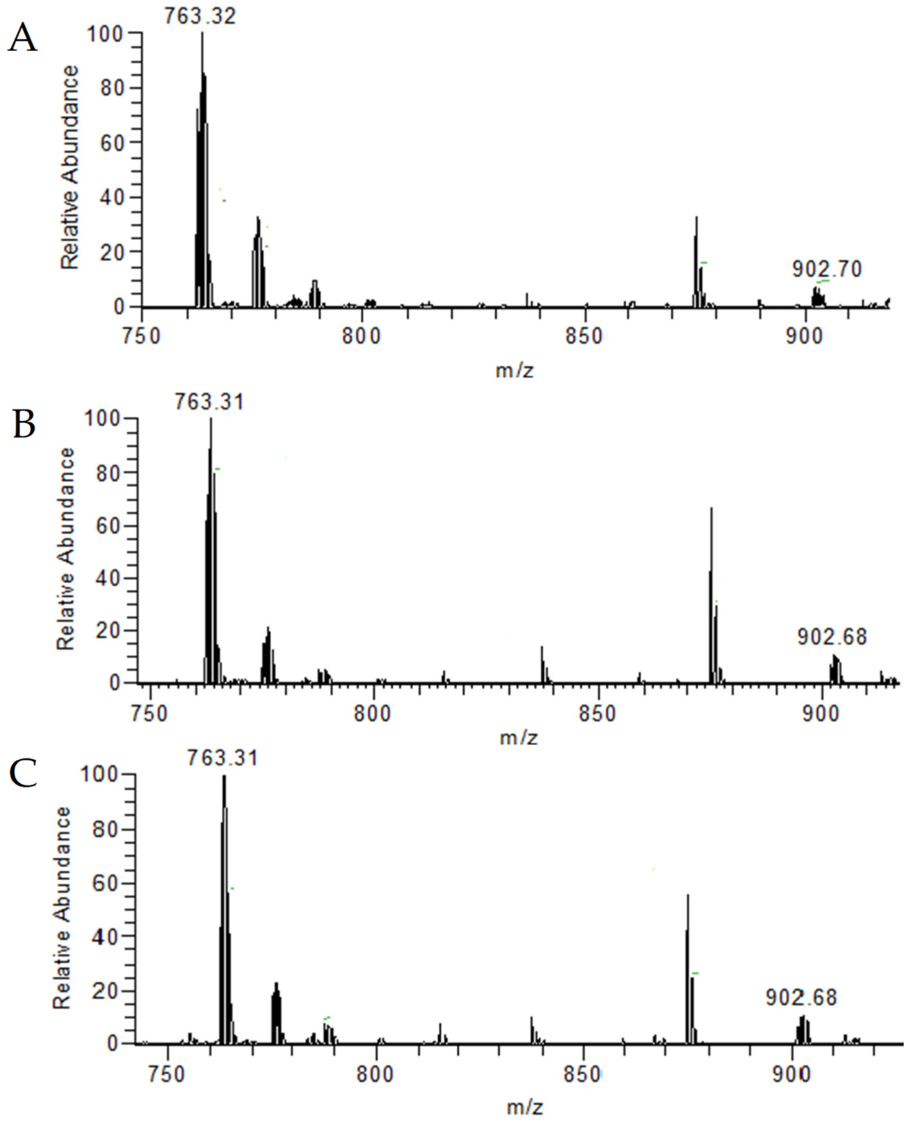

Figure S4. ESI-MS spectra of the reaction mixture between ZF and ILQ. The selected region of the spectra shows 3+ charged peaks. The reaction was performed on 10 μM ZF with 30 μM ILQ. The spectra were recorded after incubation for 15 min (A), 2h(B) and 5 h(C), respectively.

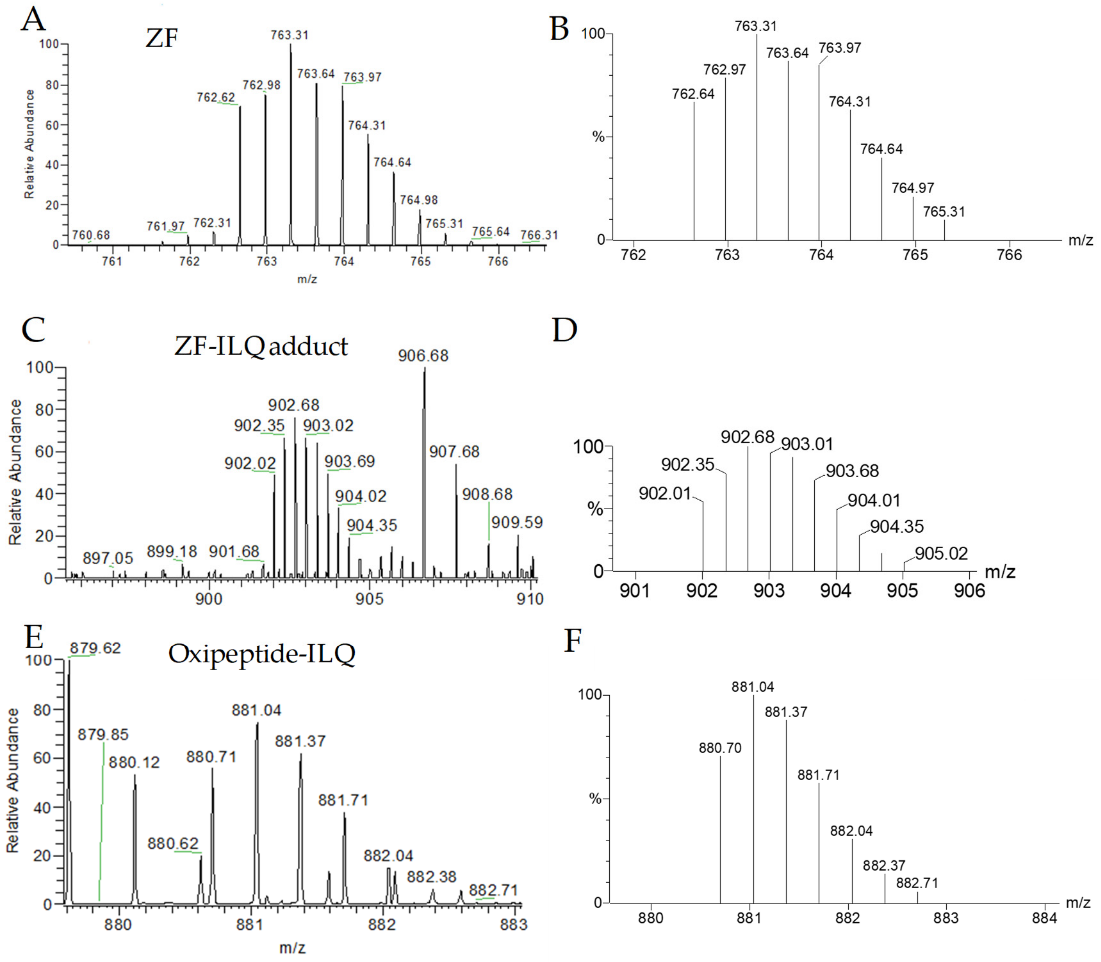

Figure 5. Species observed in ESI-MS spectrum for the reaction mixture between ZF and ILQ. Observed (A, C, E) and calculated (B, D, F) mass spectra of the species.

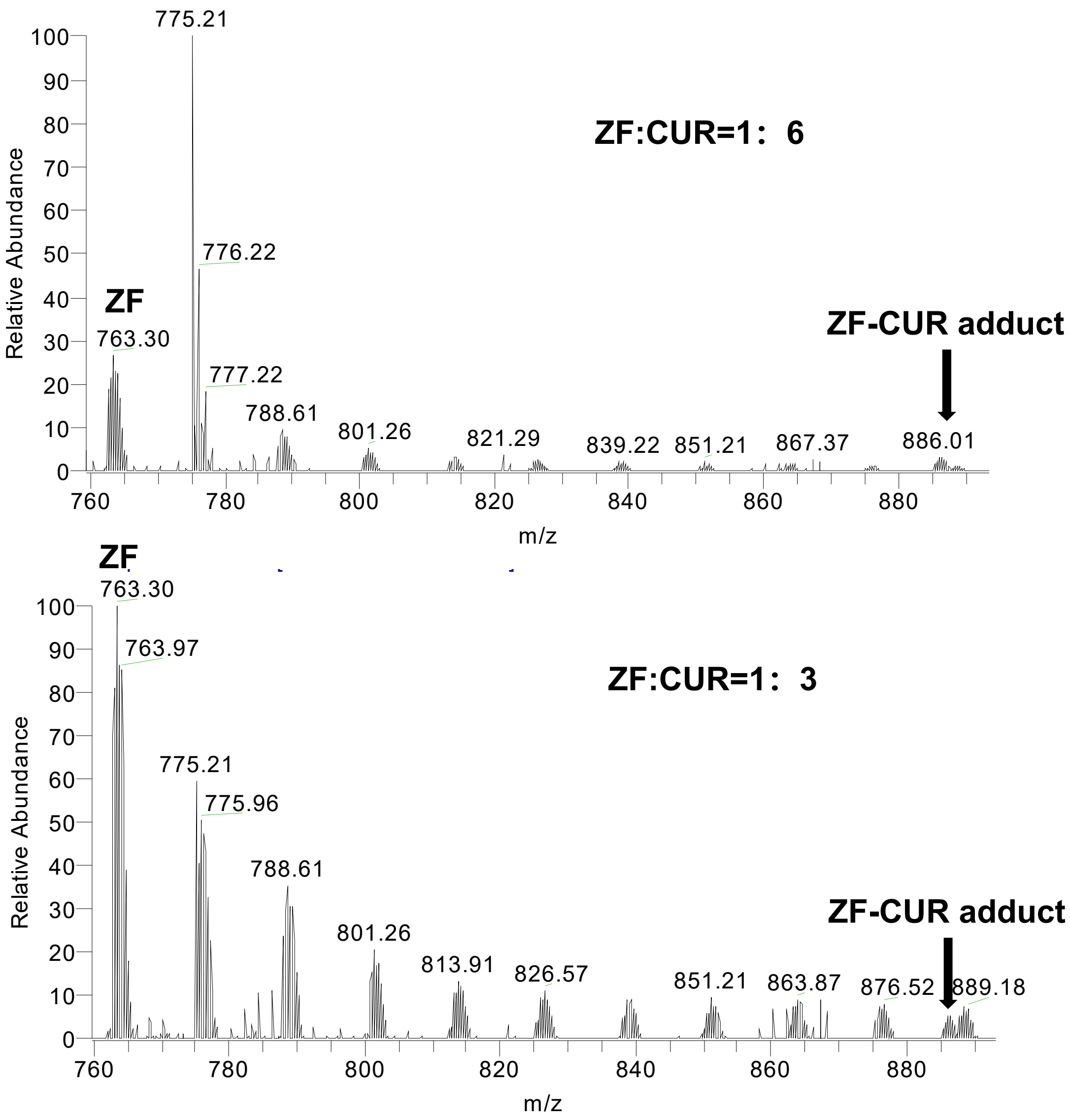

Figure S6. ESI-MS spectra of the reaction mixture between ZF and CUR. 10 μM ZF was incubated with 60 μM CUR for 15 min (top). 10 μM ZF was incubated with 30 μM CUR for 15 min (bottom).

/

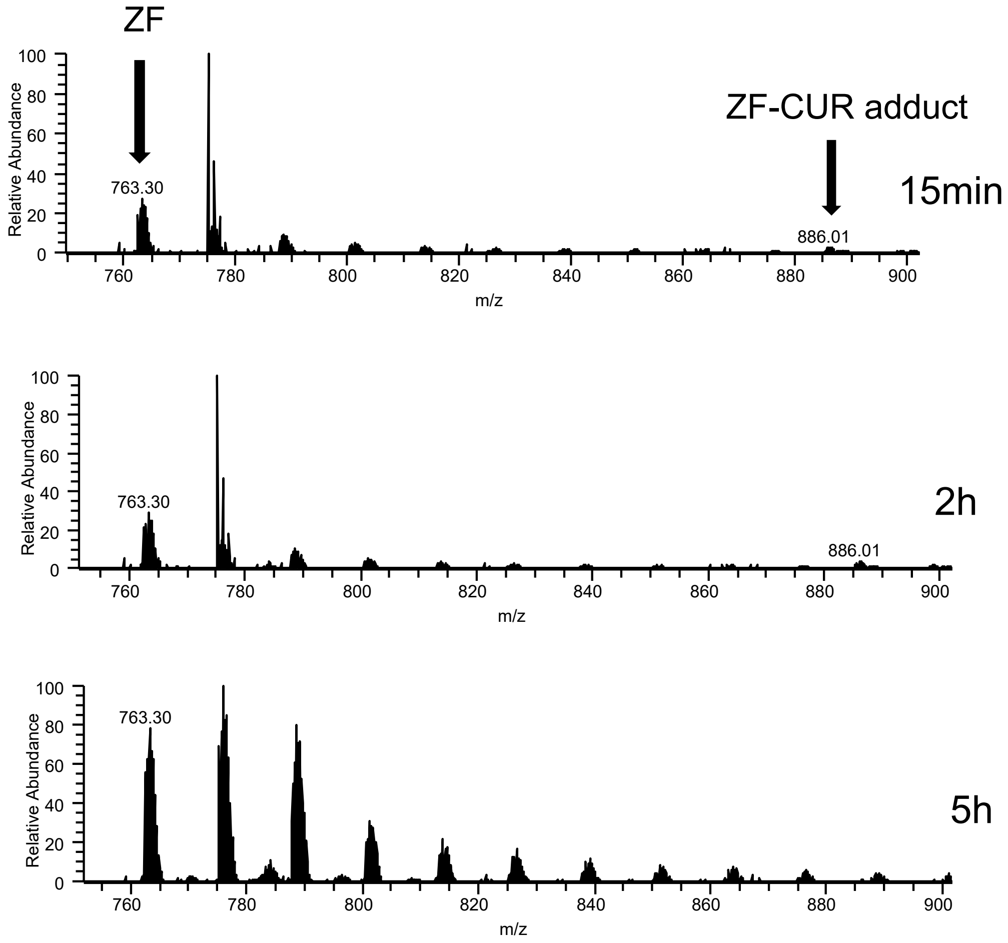

Figure S7. ESI-MS spectra of the reaction mixture between ZF and CUR. The selected region of the spectra shows 3+ charged peaks. The reaction was performed on 10 μM ZF with 60 μM CUR. The spectra were recorded after incubation for 15 min, 2h and 5 h respectively.

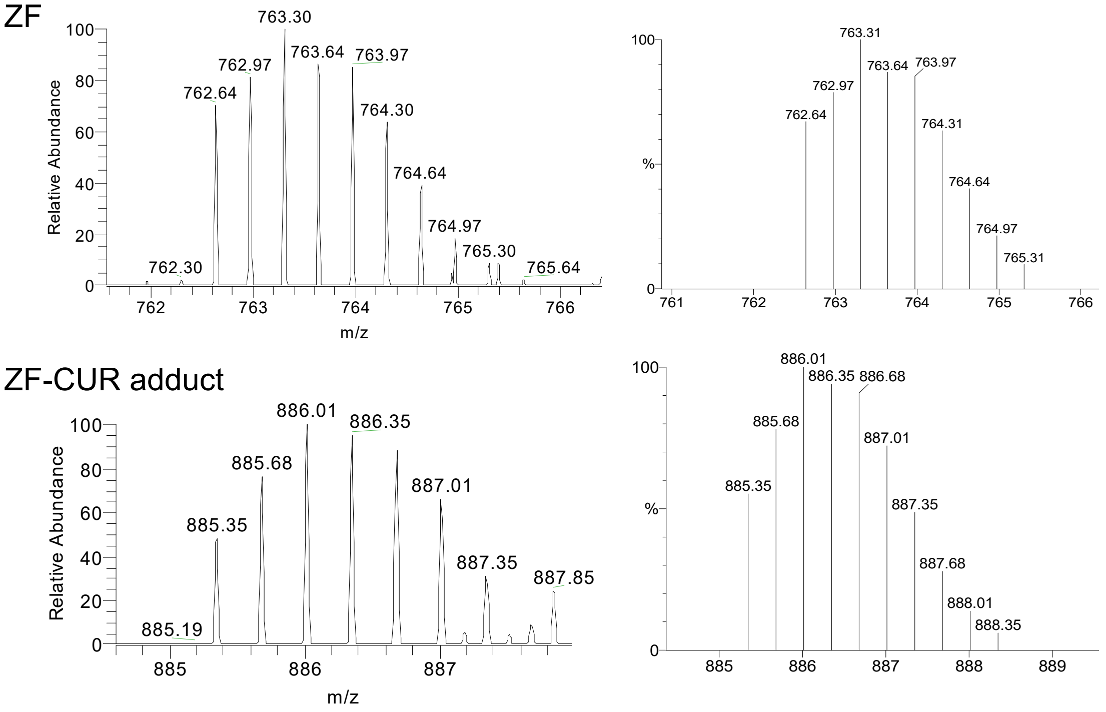

Figure S8. Species observed in ESI-MS spectrum for the reaction mixture between ZF and CUR. Observed (left) and calculated (right) mass spectra of the species.
